# (v) Simulation and measurement of wear in metal-on-metal bearings *in vitro*- understanding the reasons for increased wear

**DOI:** 10.1016/j.mporth.2012.05.005

**Published:** 2012-08

**Authors:** John Fisher, Mazen Al Hajjar, Sophie Williams, Joanne Tipper, Eileen Ingham, Louise Jennings

**Affiliations:** **John Fisher CBE BSc PhD DEng** Professor, Institute of Medical and Biological Engineering iMBE, University of Leeds, Leeds, UK and Leeds Musculoskeletal Biomedical Research Unit, Leeds Teaching Hospital Trust, Leeds, UK; **Mazen Al Hajjar BEng** Research Engineer, Institute of Medical and Biological Engineering iMBE, University of Leeds, Leeds, UK and Leeds Musculoskeletal Biomedical Research Unit, Leeds Teaching Hospital Trust, Leeds, UK; **Sophie Williams BSc PhD** Senior lecturer, Institute of Medical and Biological Engineering iMBE, University of Leeds, Leeds, UK and Leeds Musculoskeletal Biomedical Research Unit, Leeds Teaching Hospital Trust, Leeds, UK; **Joanne Tipper BSc PhD** Senior lecturer, Institute of Medical and Biological Engineering iMBE, University of Leeds, Leeds, UK and Leeds Musculoskeletal Biomedical Research Unit, Leeds Teaching Hospital Trust, Leeds, UK; **Eileen Ingham BSc PhD** Professor, Institute of Medical and Biological Engineering iMBE, University of Leeds, Leeds, UK and Leeds Musculoskeletal Biomedical Research Unit, Leeds Teaching Hospital Trust, Leeds, UK; **Louise Jennings MEng PhD** Principal Research and Innovation Fellow, Institute of Medical and Biological Engineering iMBE, University of Leeds, Leeds, UK and Leeds Musculoskeletal Biomedical Research Unit, Leeds Teaching Hospital Trust, Leeds, UK

**Keywords:** adverse conditions, edge loading, metal-on-metal hips, pre-clinical simulation, stratified approach for enhanced reliability SAFER, wear

## Abstract

A new Stratified Approach For Enhanced Reliability (SAFER) pre-clinical simulation testing of joint prostheses has been described in a preceding paper in this volume. The application of SAFER *in vitro* simulation and testing to metal-on-metal bearings is described in this review paper. The review aims to provide further understanding of the reasons for, and causes of, increased wear in metal-on-metal hips in a proportion of patients. Variation in positioning (mal-positioning) of the head and cup in hip prostheses results in the head contacting the rim of the cup and producing increased wear. Variation in both translational and rotational positioning has been investigated. Variation in translational positioning of the centres of the head and cup, which is not detected on radiographs, is a frequent occurrence clinically and can result in a substantial increase in wear rate. The variation in translational positioning acts synergistically with variation in rotational positioning to produce substantial increases in wear. These recent findings are consistent with the wear mechanisms and formation of stripe wear reported for ceramic-on-ceramic bearings over a decade ago, and provide insight into the reasons for the variation and increases in the wear rate found clinically in metal-on-metal hips in specific patients, which may cause premature failure.

## Introduction

Metal-on-metal bearings for hip prostheses, manufactured from cobalt chromium alloys, were first used clinically in the 1960s. While some individual prostheses showed long survivorship,^1^ they did not gain widespread adoption. During the 1970s, 1980s and 1990s, the majority of hips implanted used metal-on-polyethylene bearings. The onset and awareness of polyethylene wear debris-induced osteolysis^2^ in the 1990s, led to a renewed interest in metal-on-metal hip prostheses. Initially metal-on-metal bearings were introduced in total joint replacements with small or medium size heads, with diameters of 28, 32 or 36 mm. They were subsequently introduced as bearings with larger diameter heads (>36 mm) in surface replacement hips, which were able to preserve bone on the femoral side. The potential advantages of larger sized bearings, with an increased range of motion and greater stability, prompted the introduction of larger diameter metal-on-metal bearings (>36 mm) in total hip replacements.

There has been considerable publicity and clinical literature published regarding the adverse effects of higher than expected metal wear rates and ion release, and poor clinical results with large diameter metal-on-metal bearings. While many of these clinical reports define clinical failure or revision rates, they can only speculate about the causes of high wear. The causes of high wear, metal ion release, adverse tissue reactions and failure are multi-factorial and result from specific combinations of conditions in a complex tribological system. This review provides a basis for improved scientific understanding of the reasons for high wear in metal-on-metal bearings.

An area of recent concern, the corrosion and wear of tapers and trunnions at the interface of the large diameter metal head with the stem, is deliberately not covered in this review. This corrosion and wear predominantly occurs with large diameter metal heads being used on the taper junctions of stems designed for small diameter heads. The additional forces and torques associated with the large diameter heads can result in micro-motion, fretting, corrosion and wear at the taper junction. This will be covered in other publications.

This review focuses on metal wear and ion release from the bearing surfaces of metal-on-metal bearings. The review describes laboratory simulations and measurements of bearing wear and investigations of the causes of elevated metal wear and ion release, which can provide further insight and understanding of causes of clinical failure.

Clinical concerns about wear, ion release and adverse tissue reactions with metal-on-metal surface replacement bearings have emerged gradually over the last decade. The cellular toxicity of actual metal-on-metal wear debris, at clinically relevant doses, was first reported in 2003.^3^ In 2004, Brodner et al., reported elevated metal ion levels in metal-on-metal total hip replacements,^4^ which were associated with high cup inclination angles. This was supported by clinical studies of metal-on-metal surface replacements in 2008, which also demonstrated elevated ion levels with high cup inclination.^5^ Following earlier reports of abnormal tissue reactions in metal-on-metal hips [aseptic lymphocytic vasculitis associated lesion; ALVAL], adverse tissue reactions, described as pseudo-tumours, with the Birmingham metal-on-metal surface replacement^6^ were recorded in 2008. In addition, at this time, Morlock et al. reported edge loading and high wear in a series of metal-on-metal surface replacement retrievals, again associated with cups with high inclination angles.^7^ Since these reports were published it has become clearer that high wear and elevated ion levels are not only associated with cups implanted at high levels of inclination, but also with other forms of mal-positioning, specifically variation in the translational position of the centres of the cup and head.

During the development of surface replacement metal-on-metal bearings in the late 1990s and early 2000, some pre-clinical *in vitro* simulation testing of metal-on-metal hips was undertaken. However, this was predominantly undertaken under ISO standard conditions with a standard walking cycle and correctly positioned components. Under these conditions the wear of metal-on-metal bearings was low and less than 1 mm^3^/million cycles. Clearly these standard conditions do not adequately reflect the wider set of conditions found in the patient or the potential for conditions in the patient to cause elevated wear rates.

Since 2000, at the University of Leeds, we have developed a unique alternative “**S**tratified **A**pproach **F**or **E**nhanced **R**eliability” (**SAFER**), pre-clinical simulation and testing methodology, described earlier in this volume.^8–10^ SAFER adopts a systematic approach to pre-clinical simulation and measurement of wear under a wider set of clinical conditions, including adverse conditions which may increase wear. In this review paper, we describe how this approach has been applied to simulate and measure elevated wear *in vitro* in metal-on-metal hips, replicating the high wear and elevated metal ion levels found in patients. In particular, it describes how variation in translational position and rotational position (mal-positioning) can result in the femoral head contacting the rim of the cup, resulting in increased wear.^8^

**Key learning points:**

• Higher than expected failure rates with large diameter (>36 mm) metal-on-metal bearings in surface replacement and in total hip replacements.
• Failures associated with elevated wear of metal-on-metal bearings.
• High wear clinically associated with steeply inclined cups and other conditions that cause loading of the femoral head on the rim of cup, “edge loading.”
• High wear and edge loading is also associated with other clinical conditions.
• In total joints with large diameter metal bearings, taper or trunnion wear and corrosion on the stem has been reported, but this is excluded from this review.

## The concept of mal-positioning and edge loading – lessons learnt from ceramic-on-ceramic bearings in hip prostheses

**Figure 1:**
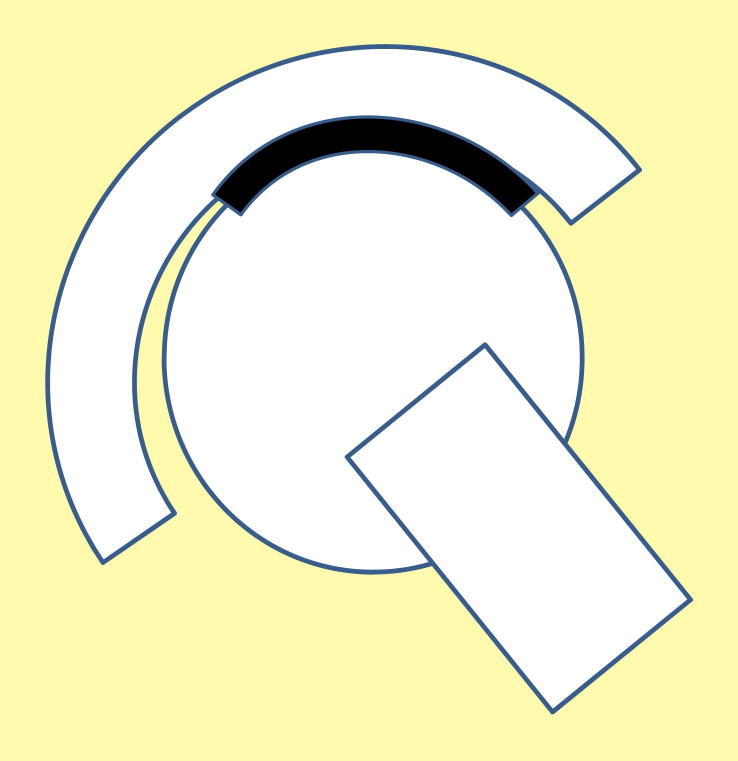
Diagram of correctly positioned hip prosthesis, with contact patch on head within the bearing area of the cup.

In correctly implanted hip joints both of the components, the head and the cup, are correctly positioned in terms of both rotation and translation. Rotational mal-positioning can be readily seen from radiographs in terms of inclination and version of the cup and the head. Translational mal-positioning is, however, less clear. The centre of the cup should be positioned at the centre of the natural acetabulum, and the centre of the head should be positioned concentric to the cup at the centre of the cup and natural acetabulum. The correctly positioned head and cup is shown in Figure 1 as a cross section in the medial lateral plane. The contact between the head and the cup is within the bearing surface of the cup.

**Figure 2:**
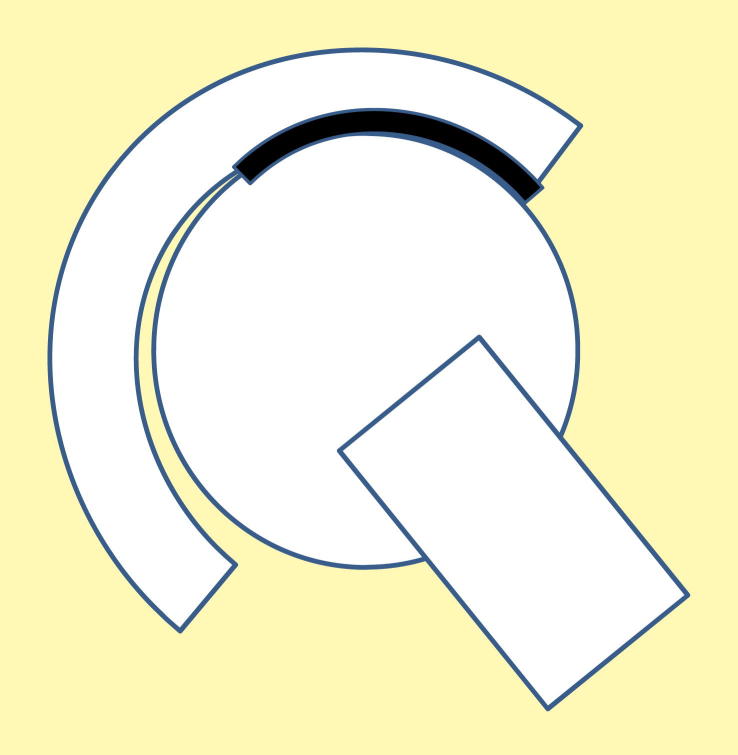
Diagram of rotational mal-position with the cup inclined at 60° and the contact patch on the head intersecting the rim of the cup.

Rotational mal-positioning of the cup is shown in Figure 2. It is represented as an increase in inclination of the cup. This rotational mal-positioning is readily seen on radiographs. In this case a steeply inclined cup results in the contact patch on the head intersecting the rim of the cup (edge loading).

**Figure 3:**
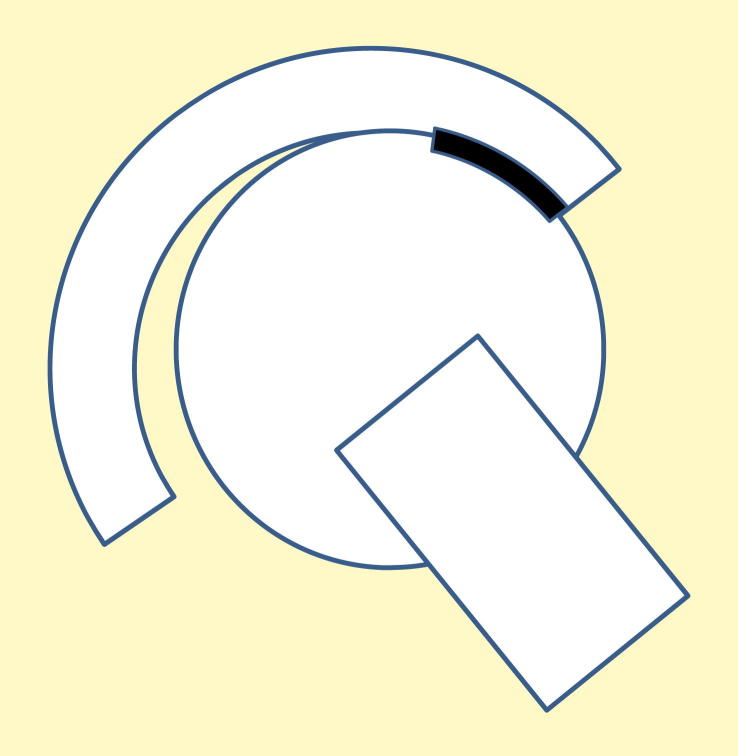
Diagram of translational mal-position with the cup medialized by 0.5 mm from the centre of the head, resulting in the contact patch of the head intersecting the rim of the cup.

Translational mal-positioning of the head or the cup in the medial–lateral plane, which results in lack of concentricity of their centres, may result from a number of factors. These include: medialization of the cup; lateralization of the head; a deficiency in the femoral offset; subsidence of the stem, soft tissue laxity or impingement. This is shown schematically in Figure 3 in which the contact zone on the head intersects the rim of the cup. In this diagram the clearance between the head and the cup is magnified, to allow the effect of translational mal-position to be visualized. In reality, with a radial clearance between the head and cup of less than 0.5 mm, the translational mal-position is not visible on radiographs. On a radiograph, a mal-positioned femoral head may appear to be correctly positioned because the rim of the cup constrains the head into the apparently correct position. For example, if the head is mal-positioned, by an offset deficiency of say 1 mm laterally, this will not be detected as the head is contained within the radial clearance of the cup, as a result of the contact of the superior rim of the cup on the head. On a radiograph the head will appear to be correctly positioned in the cup, since the X-rays do not have sufficient spatial resolution to detect the translation of the head within the radial clearance of the cup, which is typically less than 0.5 mm. The incidence of translational mal-position has not been reported clinically since it cannot be seen on radiographs. Indeed, translational mal-position has been largely ignored in the clinical literature.

Our work on ceramic-on-ceramic bearings in 2000–2003, provided insight into the significance and frequency of translational mal-position in hip prostheses. Retrieval studies of retrieved ceramic heads revealed a pattern of “stripe wear” on the majority of explanted components which was associated with the contact with the rim of the cup, “*edge loading*”.^11–13^ These studies also showed an association of stripe wear with cups with a steeper inclination angle. Steep inclination of the cups in ceramic-on-ceramic bearing *in vitro* in the simulator, however, did not reproduce stripe wear in ceramic-on-ceramic bearings.^14^ This has been confirmed in recent studies.^15^ Most importantly the stripe wear was only replicated in ceramic bearings during *in vitro* simulation by translational mal-position of the head relative to the cup, previously described as micro-separation.^12,16^ This has also been confirmed in recent work.^15^ In addition, the bimodal size distribution of ceramic wear debris found *in vivo* was only simulated and replicated *in vitro* with translational mal-position and stripe wear.^17^ Stress analysis has revealed extremely high contact stresses in association with translational mal-positioning and micro-separation in alumina ceramic bearings.^18^ This work has led us to believe that translational mal-positioning and contact of the femoral head on rim of the cup, is a common occurrence, not only in retrievals but also in implants which have not been revised. Variation in the relative translational positioning of the centre of the head, centre of the cup and natural hip of up to 1 mm or greater is, perhaps to be expected. These findings led us to introduce translational mal-positioning into a stratified approach for simulation testing of new bearing materials from 2003 onwards.^19^

**Key learning points:**

• Components can be mal-positioned in terms of rotation and in terms of translation, translation mal-position means that the centres of the bearing are not concentric.
• Rotational mal-position is readily seen on radiographs. It is frequently discussed and reported.
• Translational mal-position is common, but is not detected on standard X-rays.
• Less than 0.5 mm translational mal-position is needed to produce contact of the femoral head on rim of the cup to produce stripe wear.
• In ceramic-on-ceramic bearings, stripe wear on the head is produced by translational mal-position, not rotational mal-position.

## The tribology, contact mechanics, lubrication, friction and wear of metal-on-metal hips

Tribology is the study of contact mechanics, lubrication, friction and wear. Metal-on-metal bearings are manufactured from cobalt chrome alloys. In other tribological systems, dry metal-on-metal bearings comprised of similar materials, result in high adhesive friction and wear. Analysis of the lubrication regime of metal-on-metal bearings in the hip,^20^ has shown that they are not lubricated by a fluid film and that direct contact of the solid metal surfaces occurs. This can result in high friction and wear. Essentially metal-on-metal bearings operate in a mixed lubrication regime, in which the level of friction and wear depends on local tribological conditions. Lubrication with water results in much higher friction and wear compared to lubrication with a protein-containing serum solution. Friction has been shown to decrease with increased protein concentration.^21^ It is believed that the proteins in the serum form a carbon-rich boundary layer that protects against wear and corrosion.^22,23^ Evidence that the wear of metal-on-metal bearings is dependent on this boundary lubrication layer, is provided by measurement of higher wear rates during the bedding-in phase of wear,^24,25^ as the geometries of the head and cup become matched and the boundary layer is formed. Further evidence of the dependence of the wear on this lubrication regime comes from studies of the effect of the level of swing phase load on friction and wear in metal-on-metal bearings. An increase in swing phase load from 100 N to 300 N, has been shown to cause an increase in both friction and wear.^26^

Following on from our work on ceramic-on-ceramic bearings, we carried out the first ever studies of the effects of translational mal-position and edge loading on friction and wear of metal-on-metal bearings.^27^ This showed a marked increase in wear and large variations in wear rates, when the metal head contacted the rim of the metal cup.^28^ This, along with our experience with ceramic-on-ceramic bearings motivated us to undertake a longer series of further studies of the wear of metal-on-metal bearings under rotational and translational mal-position, which we report in this review.

**Key learning points:**

• Metal-on-metal bearings are a lubrication sensitive tribological system. Low wear is achieved by a protein boundary layer which reduces friction and wear.
• Low wear is achieved under standard walking conditions with correctly aligned components, once the boundary layer is formed by entrainment of the lubricant.
• Higher wear occurs in the initial bedding in phase.
• Higher wear will occur when there are elevated stress states which prevent the formation of a boundary layer, such as in edge loading of the head on the rim of the cup.

## Methods of simulation and measurement of wear in metal-on-metal hips *in vitro* with translational and rotational mal-position

In this review we report on *in vitro* wear simulator studies of size 36 mm metal-on-metal bearings for total hip joints with a full hemisphere acetabular cup, and size 39 mm surface replacement bearings with a sub hemisphere acetabular cup. At least three components of each type were studied. The wear simulation was carried out under three conditions:

• Ideal walking cycle with correctly positioned components as per ISO standard.
• Rotational mal-position with the acetabular cup positioned between 55° and 65°.
• Translational mal-position with micro-separation of 0.5 mm.

Full details of the simulation methodologies and methods of measurement are given in previous publications.^28,29^

**Key learning points:**

• Rotational mal-positioning of the cup at a steep inclination angle of 55–65° has been simulated *in vitro* to replicate one type of edge loading of the femoral head on the rim of the cup.
• Translational mal-position of the head with respect to the cup, by 0.5 mm medialization of the cup with respect to the centre of the head, has been simulated *in vitro* to replicate variation in translational positioning found clinically, a second type of edge loading of the femoral head on the rim of the cup.
• Both translational and rotational mal-position have been simulated together.

## Results, measurement of wear in hip joint simulators *in vitro* under adverse conditions with translational and rotational mal-position

For the full hemisphere 36 mm metal-on-metal bearing, the wear rate under both standard walking conditions and conditions with rotational mal-position of 65° were low and less than 1 mm^3^/million cycles with values of 0.35 and 0.37 mm^3^/million cycles respectively. No increase in the wear or stripe wear or loading on the rim of the cup was found at 65° rotational mal-position.

For the sub-hemispherical 39 mm surface replacement, low wear of less than 1 mm^3^/million cycles was found under standard conditions, but for rotational mal-position, stripe wear and an increased wear rate of 4.5 mm^3^/million cycles was found, which was more than 20 times higher than the wear rate under standard walking conditions.

**Figure 4:**
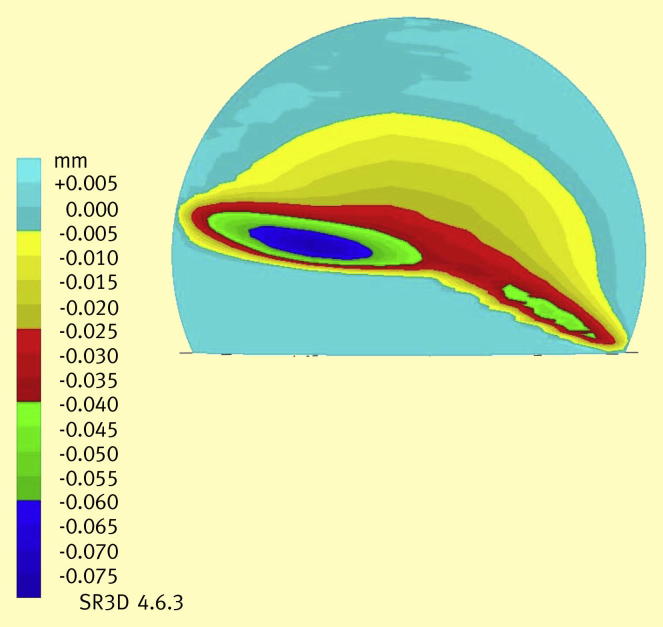
Three dimensional co-ordinate measurement of the 36 mm femoral head after translational mal-position showing high stripe wear on the head, where it has contacted the rim of the cup.

For translational mal-position and micro-separation, stripe wear and elevated wear rates were found for both prostheses. For the 36 mm prostheses stripe wear rates of approximately 4.7 mm^3^/million cycles were found. An example of the stripe wear on a 36 mm head is shown in Figure 4. For the 39 mm sub-hemispherical surface replacement a higher stripe wear rate of 9.5 mm^3^/million cycles was found. This wear rate was more than 50 times higher than the wear rate than found under standard walking conditions. These severe wear mechanisms were also found to generate larger wear particles up to 1000 nm in size, compared to the wear particles in 10–30 nm sized range under standard walking conditions, replicating the range of wear particles found clinically.

**Key learning points:**

• Standard walking cycle simulation with correctly positioned components produced low wear rates, which are unlikely to cause adverse tissue reactions in the patient.
• Rotational mal-positioning of a full hemispherical cup inclined at 65° also produced a low wear rate.
• Rotational mal-position of a sub-hemispherical cup at 60° inclination produced a 20 fold increase in wear rate, stripe wear on the head and wear on the rim of the cup.
• Translational mal-position (micro-separation) produced increased stripe wear on both the full hemispherical and sub-hemispherical cups, with stripe wear on the head and rim wear on the cup. In the case of the sub-hemispherical cup the wear rate increased by more than 50 fold compared to the standard walking cycle condition.

## Discussion

The tribology, friction, lubrication and wear of hip replacements in patients are complex, highly variable and dependent upon the wide variation in the conditions inherently found in different patients. No one single standard pre-clinical test can replicate these complex and highly variable conditions. Our stratified approach “SAFER,” to pre-clinical testing of hip replacements, provides a systematic framework in which to undertake pre-clinical studies. This review has described how this approach has been applied to metal-on-metal bearings for hip prostheses, and how it has been able to simulate the conditions that replicate the high wear rates and wear mechanisms found in some patients *in vivo*.

Under standard walking conditions, with correctly positioned prostheses, the wear rate of the metal-on-metal prostheses studied was low, less than 1 mm^3^/million cycles. This was similar to the low wear rates reported in other studies.^24,30,31^ These low levels of wear under standard conditions are not expected to result in adverse tissue reactions or failure, and formed the basis for pre-clinical testing of metal-on-metal hip joints prior to 2004.

Rotational mal-positioning is the most easily detected form of surgical mal-positioning since it can be visualized by radiographs. It is widely reported and can be exemplified by a steeply inclined cup (Figure 2). In our *in vitro* studies with an inclination angle of 60–65°, the sub-hemispherical 39 mm bearing produced increased stripe wear with the head contacting the rim of the cup, but under the same conditions, the 36 mm full hemispherical cup did not produce an increase in wear rate or stripe wear. In a separate study, unpublished results, a smaller diameter size 28 mm also produced increased stripe wear, with the head contacting the rim of the cup. Therefore, the angle of inclination at which stripe wear occurs depends on the cup design as well as the head diameter. Our previous studies on ceramic-on-ceramic bearings showed that neither 28 mm nor 36 mm bearings produced stripe wear when tested at the high cup inclination angle, implying this harder material combination is potentially more resistant to stripe wear under edge loading associated with rotational mal-position alone.

Translational mal-positioning results from a lack of concentricity of the femoral head and cup. It is a common occurrence clinically. However it is not seen on X-ray, since the rim of the cup constrains the head within the translational space defined by the radial clearance (<0.5 mm). As a result it is not readily detected clinically. It does, however, result in the head contacting the rim of the cup and results in stripe wear. In ceramic-on-ceramic bearings, translational mal-positioning occurs in the majority of patients and results in stripe wear on the head and a bimodal distribution in the size of the wear particles.^11–15^ In all metal-on-metal bearings, translational mal-positioning was shown to cause a substantial elevation of wear, and the increase in wear was greater in the sub-hemispherical cups. Rotational mal-position alone caused increased wear in sub-hemispherical cups. The extent and frequency of translational mal-position clinically will depend on surgical technique as well as the design of the prosthesis and the surgical instrument set.

The variation in surgical positioning of the centres of the components, the translational mal-position, is a significant factor in determining the wear rate of metal-on-metal bearings. This variation is not detected by radiographs and as a result is not reported in clinical or retrieval studies.

Our consideration and application of a stratified approach for increased reliability, SAFER, *in vitro* simulation and testing, which takes into account variation in surgical positioning has provided great insight into the causes of high wear in metal-on-metal hips.

**Key learning point:**

• Translational mal-positioning causes increased wear in metal-on-metal bearings in hip prosthesis.

## Summary

New SAFER (Stratified Approach For Enhanced Reliability) *in vitro* simulation methodologies have been defined and applied to metal-on-metal bearings for hip prostheses. Variations in positioning (mal-positioning) of heads and cups in hip prostheses result in the head contacting the rim of the cup and producing increased wear. Variation in translational positioning of the centres of the head and cup which are not detected on radiographs, are a frequent occurrence clinically and result in a substantial increase in wear rate.

## Statement of commercial interest

JF and SW are paid consultants to DePuy.

JF and the University of Leeds receive royalty income from DePuy.

JF and EI are share holders and paid advisers to Tissue Regenix Group plc.

Research funding has been received from the following companies in the last 5 years: DePuy, Mathys, JRI, Ceramtec, Eurocoatings, Neu Biomechanics, Biomet, Simulation Solutions, Smith and Nephew.
